# Supplementary figures and images for: Global Profiling of the Lysine Crotonylome in Different Pluripotent States
Source: Genomics Proteomics Bioinformatics. 2021 Mar 19;19(1):80–93. doi: 10.1016/j.gpb.2021.01.004 (PMC8498919; doi:10.1016/j.gpb.2021.01.004)

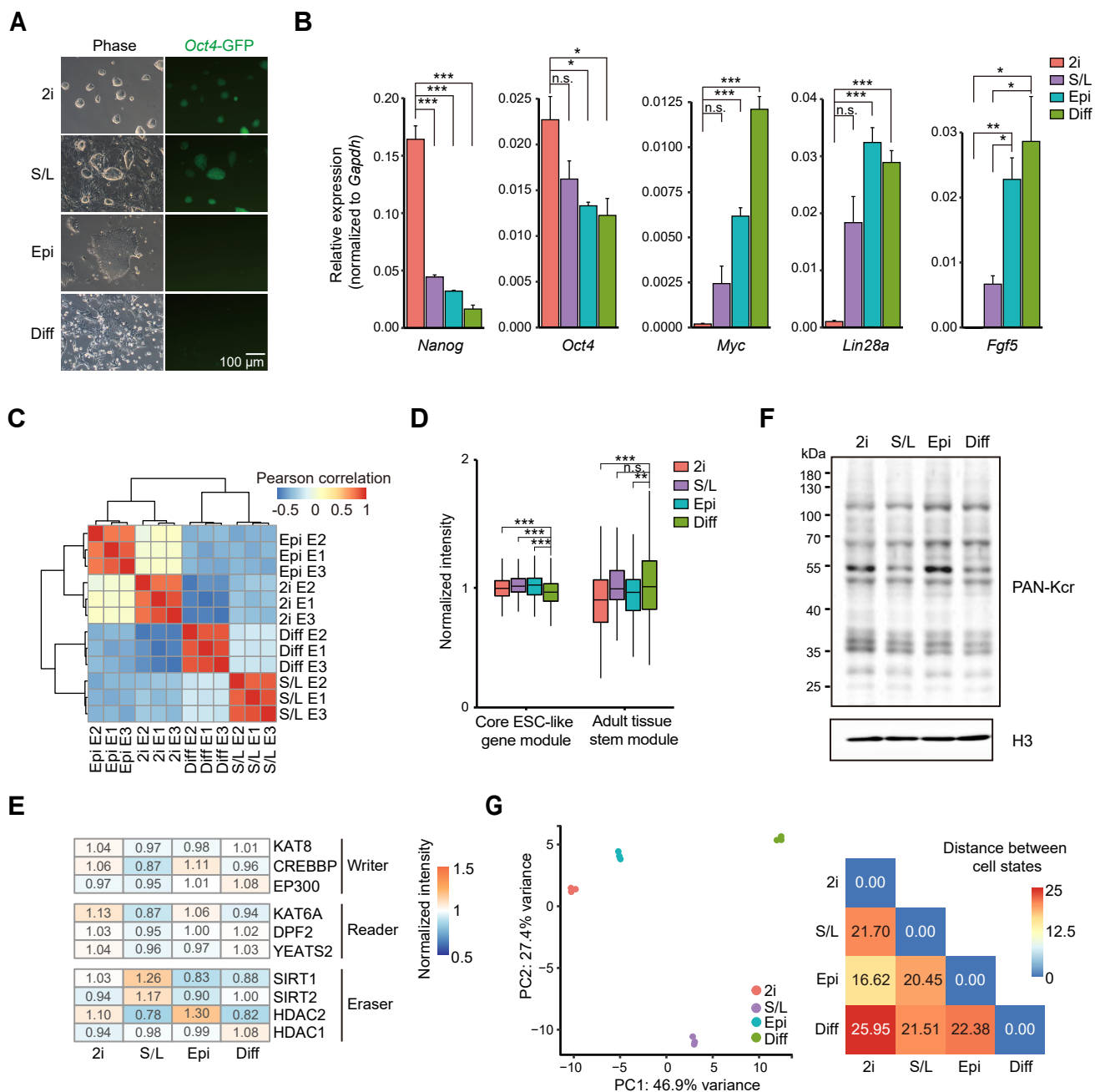

Supplement: Supplementary Figure S1 — Characterization of four different cell states A. Representative images showing PSCs cultured in different conditions, bright-field (phase) and green fluorescence for the same fields are shown. Scale bar = 100 µm. B. Relative expression of selected pluripotency and differentiation genes measured by RT-qPCR in the four different cell states. Data are presented as mean ± S.E.M. (n = 3 biological replicates with 3 technical replicates each). Gapdh was used as the housekeeping control gene. C. Hierarchical clustering of the normalized log2 intensity for the total proteome of three different LC-MS/MS experiments (biological replicates) in four different cell states, colours in the heatmap indicate pairwise Pearson correlation between the different datasets (n = 4728). D. Normalized log2 intensity of ‘Core ESC-like gene module’ and ‘Adult tissue stem module’ gene sets from MSigDB in the four different cell states (n = 3 biological replicates). E. Heatmap showing the protein expression levels of crotonylation regulators in our total proteome analysis. F. Western blotting showing the global protein crotonylation levels in the four different cell conditions. G. Left: PCA of the total proteome in the four different cell states is shown; the variance was calculated using normalized log2 intensity. Right: Euclidean distances of proteome profiles between the indicated cell conditions. *P < 0.05; **P < 0.01; ***P < 0.001; n.s., not significant (two-tailed unpaired Student’s t-test). PSC, pluripotent stem cell; MSigDB, Molecular Signatures Database. [file mmc1.pdf]

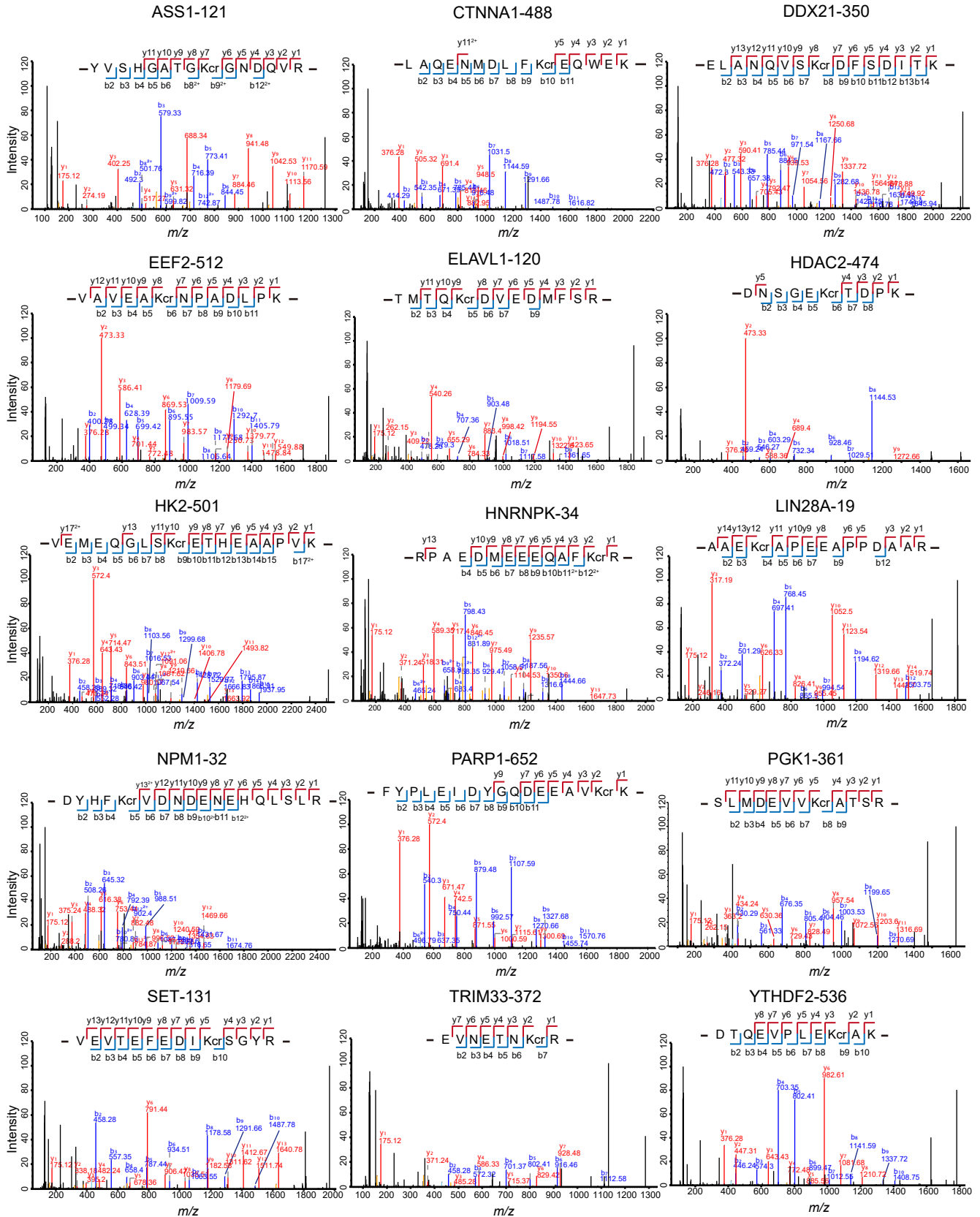

Supplement: Supplementary Figure S2 — MS/MS spectrum of identified Kcr peptides of the 15 Western blotting-validated proteins inFigure 2D [file mmc2.pdf]

**A**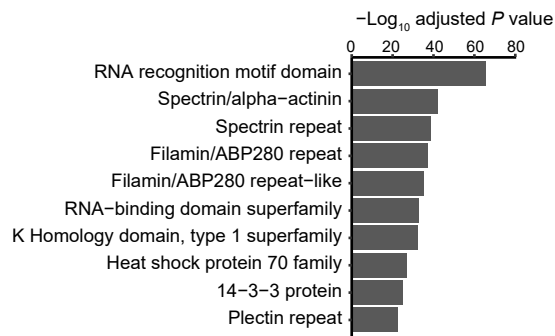**B**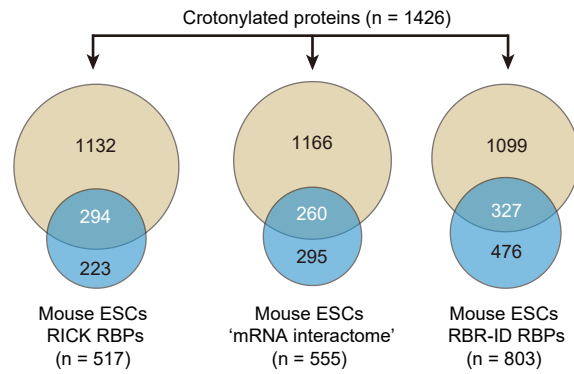**C**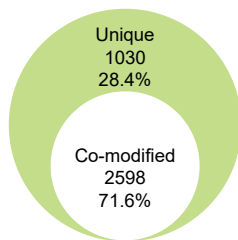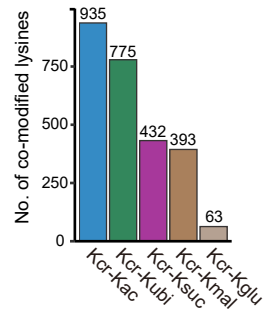**D**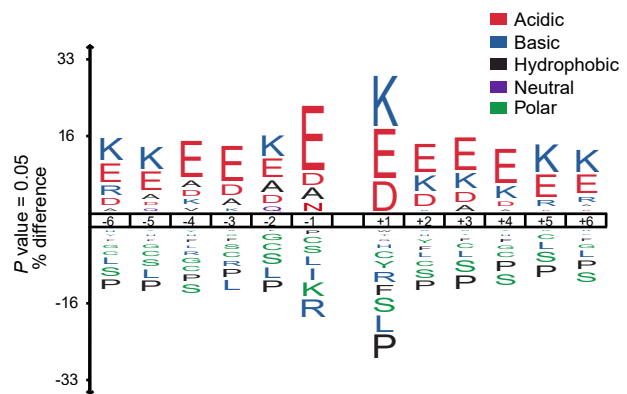**E**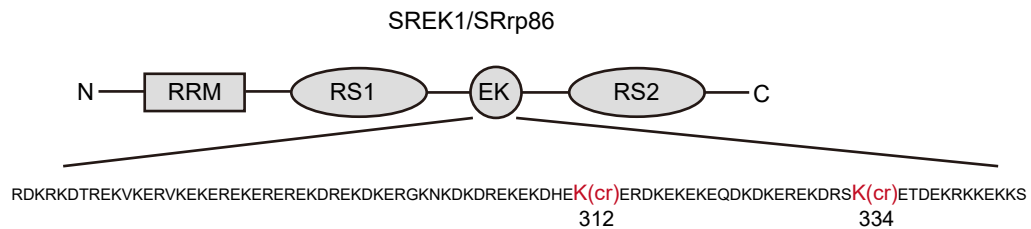**F**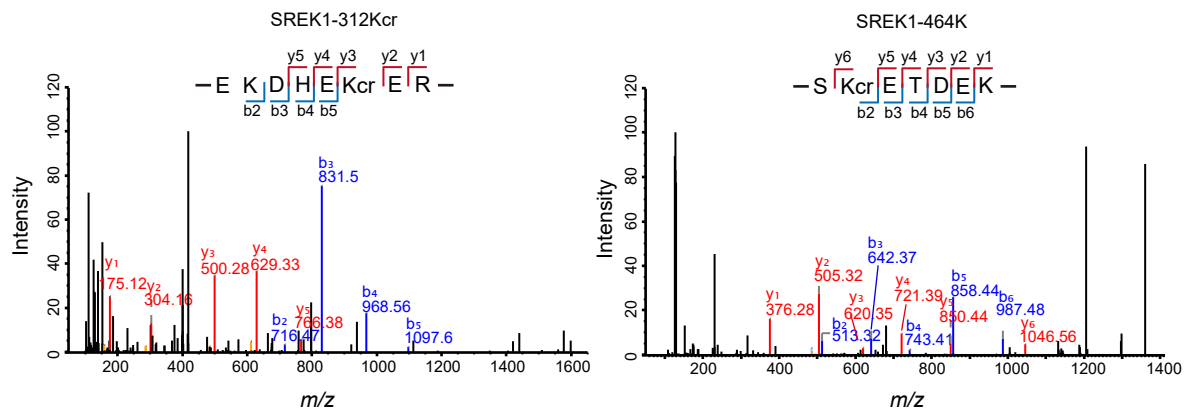

Supplement: Supplementary Figure S3 — Functional characterization of the crotonylome in the four different cell states A. Protein domain enrichment analysis of the high-confidence crotonylated proteins, the top 10 terms with the smallest adjusted P value are shown (Fisher’s exact test, Benjamini-Hochberg corrected P < 0.01). B. Venn diagrams comparing our crotonylated datasets with published mouse ESCs RBP datasets. C. Comparison of the numbers of the overlapped lysine sites between our high-confidence crotonylome and other lysine modification datasets in the PLMD database. Ac: acetylation; ubi: ubiquitination; suc: succinylation; mal: malonylation; glu: glutarylation. D. Consensus sequence motif extracted from all high-confidence Kcr sites in our datasets. E. Schematic showing lysine crotonylation sites in the EK rich region of SREK1/SRrp86. F. MS/MS spectrum of K321cr (left) and K334cr (right) for SREK1/SRrp86. PLMD, Protein Lysine Modifications Database. [file mmc3.pdf]

**A**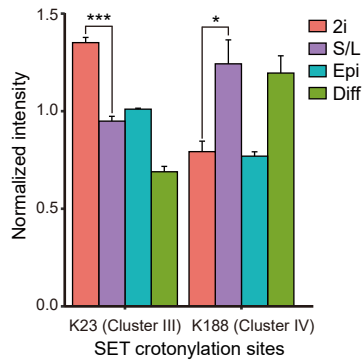**B**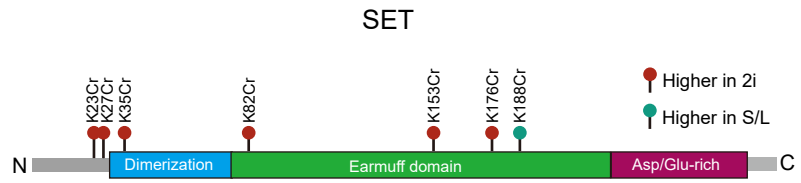**C**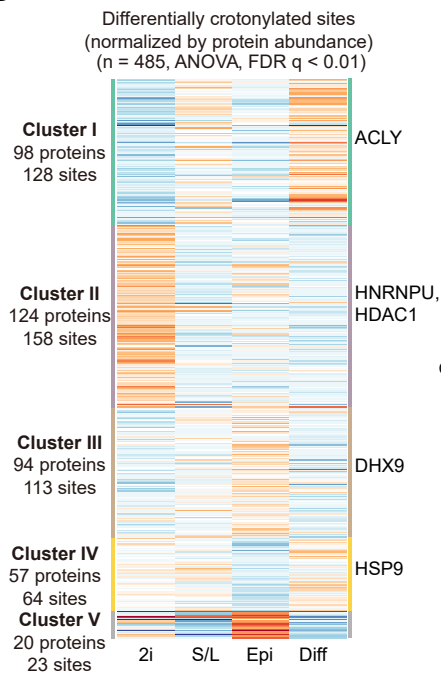**D**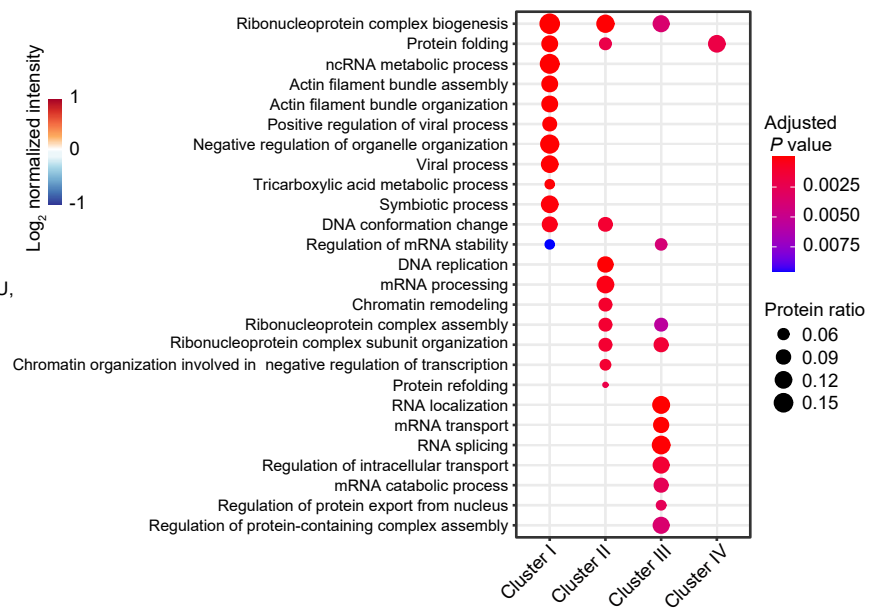

Supplement: Supplementary Figure S4 — Crotonylation of SET protein in different cell states A. Crotonylation levels of two lysine sites of SET protein in the four different cell states. Data are presented as the mean ± S.E.M. (n = 3 biological replicates, two-tailed unpaired Student’s t-test). *P < 0.05, and ***P < 0.001. B. Schematic showing the high-confidence Kcr sites of SET. Green circle: higher crotonylation level in S/L ESCs than in 2i ESCs; red circle: higher crotonylation level in EpiSCs than in 2i ESCs. C. Heatmap of differential crotonylated sites in the four different cell states after being normalized by protein abundance (n = 485, ANOVA test, FDR q < 0.01). D. GO biological process analysis for the different clusters of proteins in C (Fisher’s exact test, Benjamini-Hochberg corrected P < 0.01). [file mmc4.pdf]

## Supplementary Figure 5

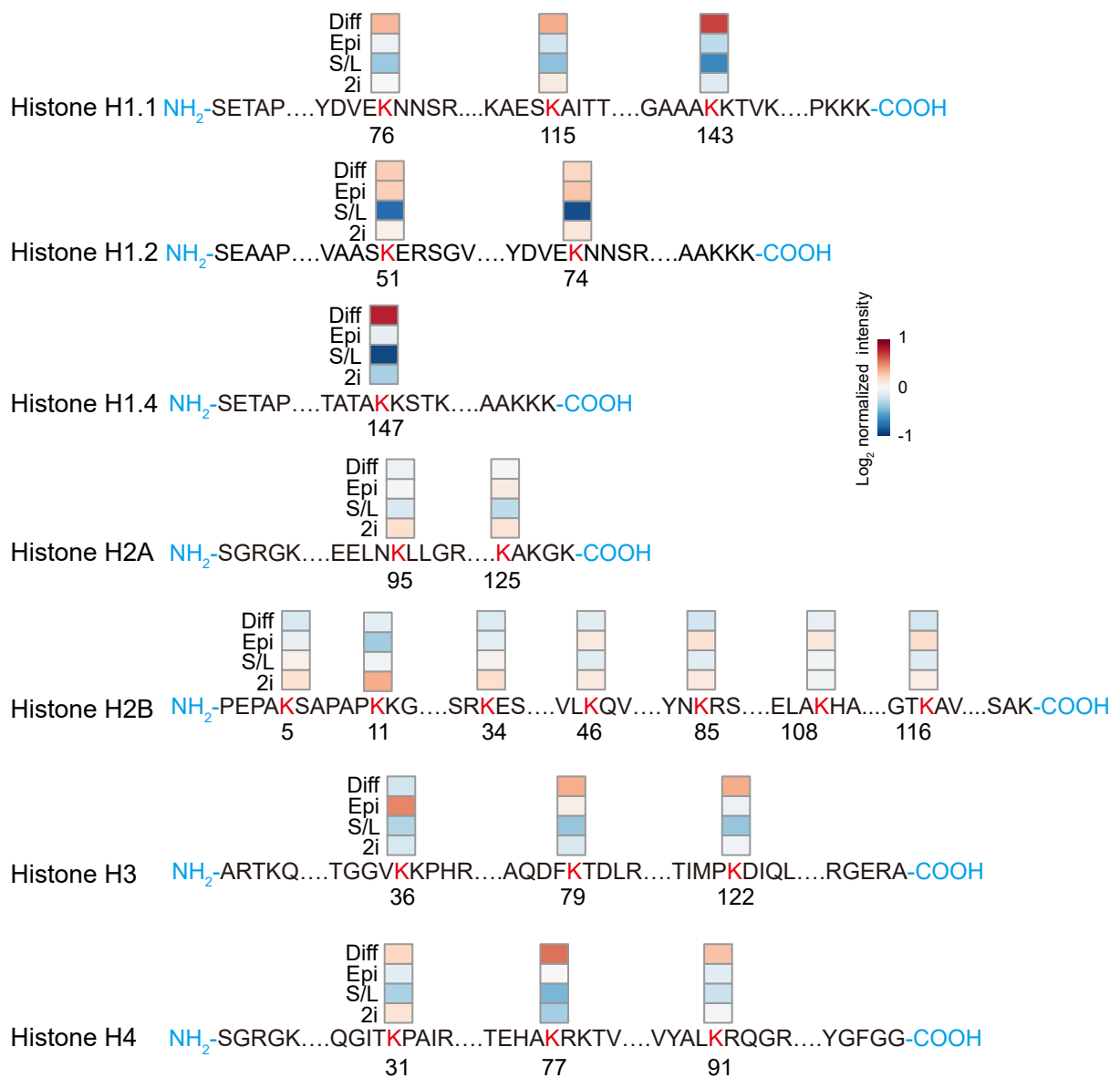

Supplement: Supplementary Figure S5 — Histone crotonylation analysis in different cell states Diagrams showing the quantitative information of histone Kcr sites in the four cell states of our study. For the isoform-specific histone sites that occurred at the same position, the mean normalized intensity has been used. [file mmc5.pdf]

**A**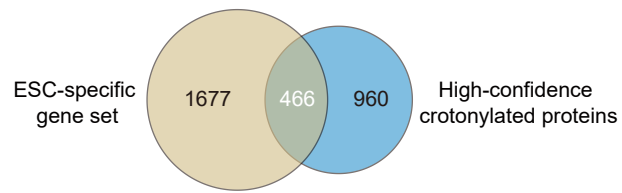**B**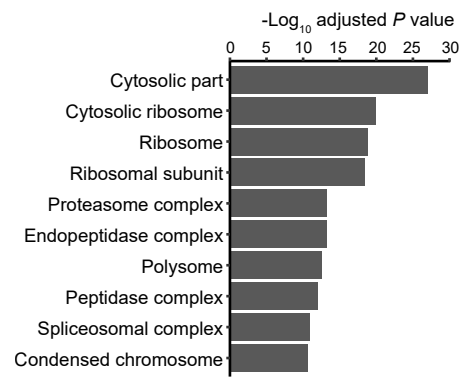**C**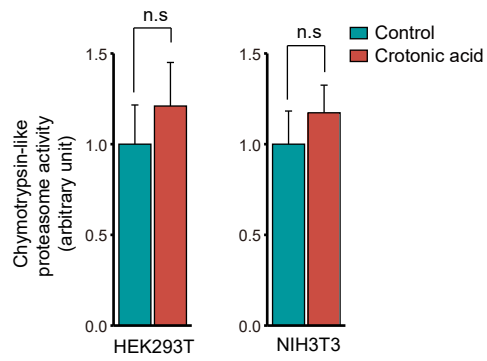

Supplement: Supplementary Figure S6 — Enrichment for ESC-specific proteins in our crotonylome A. Venn diagram comparing our crotonylome datasets with the ‘ESC-specific gene sets’. B. GO terms for the 466 overlapping proteins from (A) (Fisher’s exact test, Benjamini-Hochberg corrected P < 0.01). C. Chymotrypsin-like proteasome activity measurement of HEK293T and NIH3T3 cells with or without 10 mM crotonic acid treatment for 48 hours. (n = 3 biological replicates, two-tailed unpaired Student’s t-test). *P < 0.05; n.s., not significant. [file mmc6.pdf]
